# The matrix in focus: new directions in extracellular matrix research from the 2021 ASMB hybrid meeting

**DOI:** 10.1242/bio.059156

**Published:** 2022-01-07

**Authors:** Bryan A. Nerger, Tia M. Jones, Keron W. J. Rose, Anna Barqué, Justin S. Weinbaum, Ryan J. Petrie, Joan Chang, Davy Vanhoutte, Kendra LaDuca, Dirk Hubmacher, Alexandra Naba

**Affiliations:** 1 John A. Paulson School of Engineering and Applied Sciences, Harvard University, Cambridge, MA 02134, USA; 2 Wyss Institute for Biologically Inspired Engineering, Harvard University, Boston, MA 02115, USA; 3 Department of Biology, Drexel University, Philadelphia, PA 19104, USA; 4 Leni & Peter W. May Department of Orthopedics, Icahn School of Medicine at Mount Sinai, New York, NY 10029, USA; 5 Department of Physiology and Biophysics, College of Medicine, University of Illinois at Chicago, Chicago, Illinois 60612, USA; 6 Department of Bioengineering, Department of Pathology, McGowan Institute for Regenerative Medicine, University of Pittsburgh, Pittsburgh, PA 15219, USA; 7 Wellcome Centre for Cell-Matrix Research, Division of Cell Matrix Biology and Regenerative Medicine, Faculty of Biology, Medicine & Health, University of Manchester, Manchester M13 9PT, UK; 8 Department of Pediatrics, University of Cincinnati, Cincinnati Children's Hospital Medical Center, Cincinnati, OH 45229, USA; 9 American Society for Matrix Biology, Rockville, MD 20852, USA

**Keywords:** Microenvironment, Cell-ECM interactions, Bioengineering, Career development, Diversity, Equity, and Inclusion, Virtual meeting

## Abstract

The extracellular matrix (ECM) is a complex assembly of macromolecules that provides both architectural support and molecular signals to cells and modulate their behaviors. Originally considered a passive mechanical structure, decades of research have since demonstrated how the ECM dynamically regulates a diverse set of cellular processes in development, homeostasis, and disease progression. In September 2021, the American Society for Matrix Biology (ASMB) organized a hybrid scientific meeting, integrating in-person and virtual formats, to discuss the latest developments in ECM research. Here, we highlight exciting scientific advances that emerged from the meeting including (1) the use of model systems for fundamental and translation ECM research, (2) ECM-targeting approaches as therapeutic modalities, (3) cell-ECM interactions, and (4) the ECM as a critical component of tissue engineering strategies. In addition, we discuss how the ASMB incorporated mentoring, career development, and diversity, equity, and inclusion initiatives in both virtual and in-person events. Finally, we reflect on the hybrid scientific conference format and how it will help the ASMB accomplish its mission moving forward.

## Abstract (precis)

**Summary:** We describe recent advances and new directions in extracellular matrix research presented at the 2021 hybrid scientific meeting organized by the American Society for Matrix Biology.

## Introduction

**Fig. 1.**
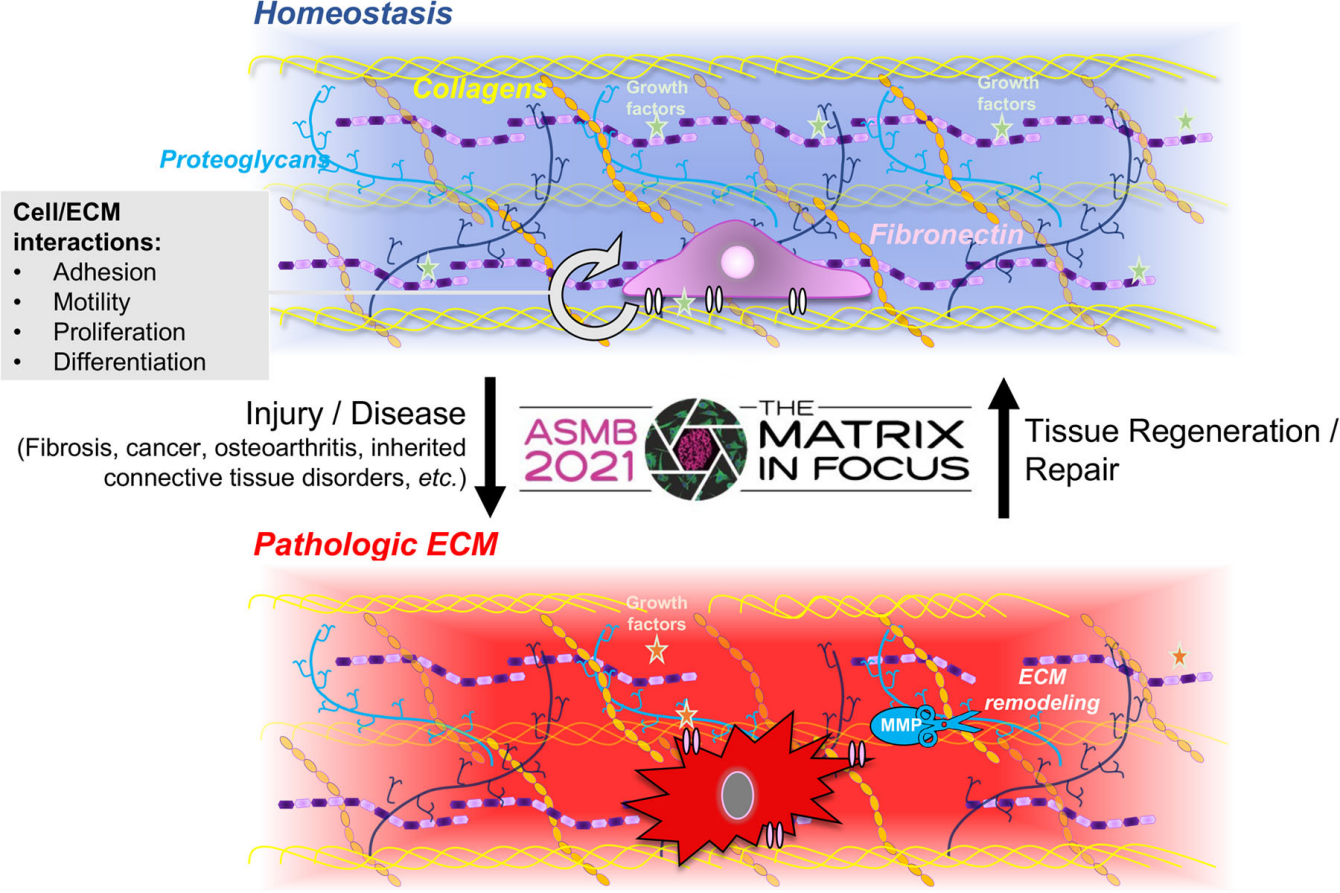
Schematic representation of the ECM in health and disease.

The extracellular matrix (ECM) is a remarkably complex assembly of hundreds of proteins and proteoglycans and a fundamental component of all multicellular organisms (Hynes and Naba, 2012; Karamanos et al., 2021). These macromolecules form a dynamic microenvironment surrounding cells and contribute biomechanical properties to connective tissues (Fig. 1). However, ECM functions extend far beyond simple structural support for cells, tissues, and organs. Over the past decades, it has become increasingly clear that the ECM, via chemical and mechanical cues, also regulates cellular phenotypes including proliferation, migration, and differentiation (Rozario and DeSimone, 2010). In addition, ECM components modulate growth factor bioavailability and signaling pathways, such as the TGFβ, BMP, or Wnt pathways (Hynes, 2009). The ECM thus plays key functional roles in tissue and organ development and homeostasis (Rozario and DeSimone, 2010). The importance of the ECM is further underscored by the fact that pathogenic variants in many genes encoding ECM protein cause disorders that can affect virtually all organ systems (Lamandé and Bateman, 2019). Prominent examples include Ehlers Danlos syndrome, Alport syndrome or Gould syndrome, which are caused by mutations in collagen genes, or Marfan syndrome and acromelic dysplasia, which are caused by mutations in fibrillin-1, ADAMTS proteases or ADAMTS-like proteins (Ramirez et al., 2018; Stanley et al., 2021). In addition, excessive degradation or accumulation of ECM is also associated with the etiology of diseases ranging from cardiovascular and musculo-skeletal diseases to cancer and fibrosis (Lu et al., 2011; Theocharis et al., 2019).

The American Society for Matrix Biology (ASMB) was founded in 2001 under the leadership of Dr. Paul Bornstein with Dr. Robert Mecham, as its first president (see https://www.asmb.net/). To advance its core mission of promoting ECM research and support development of the ECM research community, the ASMB established a biennial meeting in 2002. Originally scheduled in November 2020, ‘The Matrix in Focus’ meeting was eventually held in September 2021 in St. Louis, MO, USA, in a hybrid format that included live streaming and recording of plenary and concurrent sessions to allow for the participation of colleagues around the world unable to attend due to the COVID-19 pandemic. Guest symposia were organized by the Histochemical Society (HCS) and the Tissue Engineering and Regenerative Medicine International Society (TERMIS), emphasizing the interdisciplinary nature of ECM research, and the collaborative tradition of the ASMB. In this post-meeting report, we highlight exciting recent developments in ECM research and discuss the ASMB's experience with organizing a hybrid scientific meeting that welcomed over 350 in-person and remote participants.

## New directions in ECM research

### Model systems to advance fundamental and translational ECM research

One theme that emerged from the meeting is the incredible pace at which new model systems are being implemented to study the ECM. These new models are shedding light on unsuspected roles for the ECM in pathophysiological processes and are demonstrating how dynamic and plastic the ECM is, something that was not fully appreciated before.

Dr. Thomas Lozito (University of Southern California, Los Angeles, USA) uses lizards (*Lepidodactylus lugubris*), the closest relatives to mammals capable of re-growing appendages, to study tissue regeneration. In his presentation, Dr. Lozito showed that spinal cord-derived neural stem cells from lizards release ECM proteases, which generate a microenvironment that allows for ectopic tail growth (Lozito et al., 2021). Subsequent comparison with mouse neural stem cells showed that mammalian cells did not secrete these proteases. However, excitingly, genetic introduction of these missing proteases into mouse neural stem cells promoted lizard-like regeneration in mammals and points to a potential pathway to creating regeneration-permissive microenvironments to promote tissue restoration.

The fruit fly *Drosophila melanogaster* is a well-suited model organism to study ECM functions and dynamics, since it is amenable to high throughput functional genetic screens and has conserved ECM genes that have not undergone gene family expansions seen in vertebrates (Brown, 2011; Davis et al., 2019). Dr. Andrea Page-McCaw (Vanderbilt University, Nashville, USA) explained in her talk that basement membranes surrounding *Drosophila* gut muscles are especially sensitive to collagen IV crosslinking and other ECM regulators, suggesting active basement membrane remodeling during homeostasis (McCall et al., 2014). By damaging gut basement membranes, the Page-McCaw lab is setup to genetically identify factors required for basement membrane turnover during homeostasis and repair (Howard et al., 2019). Interestingly, most basement membrane components and regulators are critical for both repair and homeostasis, raising questions about whether there is an active form of damage detection or whether basement membrane repair is essentially a process of constant renewal.

Disease modeling using CRISPR/Cas9-mediated knock-in of patient-specific pathogenic variants in mice has become an important tool to understand the etiology of disorders caused by mutations in genes encoding ECM proteins. This is especially relevant for secreted ECM proteins, where pathogenic variants may result in dominant negative effects due to intracellular retention and therefore, straight gene knockouts may not always be appropriate for disease modeling. Dr. Douglas Gould (University of California, San Francisco, USA) studies the consequences of alterations in the basement membrane protein, collagen IV (Labelle-Dumais et al., 2019). His lab has developed a framework to understand how different variants of the same gene (or closely related genes such as *COL4A1* and *COL4A2*) can have different phenotypic consequences. Recent work from the Gould lab presented at the meeting showed that specific variants of *COL4A1* and *COL4A2* resulted in impaired secretion leading to intracellular accumulation of the proteins and triggering an endoplasmic-reticulum (ER) stress response; this in turn causes haploinsufficiency in the ECM through a loss of function or pathogenic gain-of-function of secreted and deposited collagen IV (Labelle-Dumais et al., 2019).

### ECM-targeting approaches as novel therapeutic modalities

With a better understanding of the fundamental mechanisms governing ECM homeostasis and how ECM homeostasis is disrupted in diseases, the ECM is now emerging as a ‘druggable’ target for innovative therapeutic interventions.

In the second part of his talk, Dr. Gould (see above) discussed the importance of identifying patients with *COL4A1* or *COL4A2* variants that result in the intracellular retention of these proteins, since these patients could potentially benefit from small-molecule therapeutics aimed at promoting the secretion of retained mutant proteins (Hayashi et al., 2018). Indeed, such a treatment could be effective, but only if variants of *COL4A1* and *COL4A2* result in intracellular retention without affecting protein functionality. To this aim, Dr. Gould and his team are currently mapping the functional relevance of collagen IV domains.

Pathogenic variants of other members of the collagen IV family, including *COL4A3*, *COL4A4*, *COL4A5*, can cause Alport syndrome resulting in renal failure (Funk et al., 2018). Some of these mutations generate a premature termination codon (PTC) triggering nonsense-mediated mRNA decay or resulting in truncated proteins unable to form functional collagen IV trimers. Small molecules stimulating PTC readthrough have previously been developed for muscular dystrophies or cystic fibrosis. Dr. Kohei Omachi (Washington University, St. Louis, USA) tested for the first time their efficacy on the suppression of collagen IV PTC variants. To do so, Dr. Omachi developed an *in vitro* luciferase-based screening system and a collagen-IV-assembly assay and demonstrated that some of these PTC readthrough drugs indeed increased collagen IV translation and some, but not all, *COL4A5* variants could assemble into functional trimers (Omachi et al., 2021). This opens the possibility that a subset of Alport syndrome patients may benefit from personalized treatment approaches using drugs promoting PTC readthrough.

Replacing dysfunctional ECM proteins in patients is challenging due to their large size, complicated purification and delivery protocols, and the intricate assembly machinery required to form native and functional ECM. As an alternative, gene therapy has become feasible either by correcting single nucleotide variants or by introducing functional equivalents of aberrant ECM proteins using adeno-associated viruses (AAVs) (Mendell et al., 2021). Dr. Karen McKee from the Yurchenco lab (Rutgers University, New Brunswick, USA) presented an innovative design of a ‘mini-laminin’ to ameliorate skeletal muscle dystrophy, which was small enough to be packaged by AAVs (Reinhard et al., 2017). When this construct was delivered into dystrophic mice, grip strength improved significantly. With the future use of tissue-specific promoters, it will be exciting to see how AAV-based therapies can be deployed to ameliorate connective tissue disorders in a tissue-specific and even cell type-specific manner.

### Cell-ECM interactions

The dynamic reciprocity between cells and the ECM has key implications for interdisciplinary research spanning fundamental cell biology, cancer biology, vascular biology, and bioengineering. The importance of cell-ECM interactions took center stage in the keynote address of Dr. Ken Yamada (National Institutes of Health, Bethesda, USA) who discussed the early days of cell-ECM research and reported the latest discoveries on how cell adhesion facilitates migration through three-dimensional (3D) matrices. Using particle image velocimetry, the Yamada lab measured significant deformation of the ECM in front of a fibroblast migrating through 3D collagen matrices. Using this technique, the Yamada lab identified a unique 3D migration cycle that is not predicted by observations of 2D motility (Doyle et al., 2021). Dr. Yamada also highlighted important future research goals to interrogate the molecular structure and organization of cell-matrix adhesions in 3D on curved surfaces, such as collagen fibrils, or *in vivo*.

Dr. Joan Chang from the Kadler lab (University of Manchester, United Kingdom) reported the importance of understanding the cellular machinery responsible for the trafficking of ECM proteins. The Kadler lab recently reported that the rhythmic production and assembly of ‘chrono-collagen’ provides a sacrificial ECM that protects the core ECM from daily wear-and-tear and is key to proper connective tissue function (Chang et al., 2020). In her talk, Dr. Chang examined collagen I uptake and found that cells take up exogenous collagen in a circadian manner and recycle them back into the ECM where it contributes to new fibrils, rather than simply degrading internalized collagen. She also showed that the endosome decides the fate of collagen (monomeric secretion/fibril formation), highlighting the tight cellular control over collagen fibrillogenesis and new perspectives to treating fibrotic conditions (Chang et al., 2021).

Finally, Dr. Julie DiMartino from the Bravo-Cordero Lab (Icahn School of Medicine at Mount Sinai, New York, USA) presented that the ECM does not only promote cell migration during cancer metastasis but can also convey signals fostering cancer cell dormancy. Dr. DiMartino found that a tumor-derived type-III-collagen-enriched ECM surrounds dormant tumor cell nodules. Using mouse and chicken models, she determined that downregulation of type III collagen increases metastasis and leads to straight, rather than the curly collagen fibers, an ECM phenotype associated with dormancy (Di Martino et al., 2021). These results suggest that, by manipulating ECM architecture, we may be able to induce or maintain dormancy and thus, prevent metastasis.

### The ECM: a critical component of tissue engineering strategies

The ECM has become a critical component of tissue engineering and regenerative medicine strategies, owing to its ability to regulate a diverse number of cellular processes.

Dr. Ashley Brown (North Carolina State University and the University of North Carolina at Chapel Hill, USA), recipient of the 2020 ASMB Junior Investigator award, discussed the often-overlooked importance of ECM viscoelasticity, which better describes tissue behavior under load (Chaudhuri et al., 2020), in engineering ECM microenvironments to promote tissue repair. Specifically, Dr. Brown's group found that viscoelasticity increased after injury and decreased in fibrotic tissue. Using microgels of defined viscoelastic properties, Dr. Brown identified a reciprocal relationship where cell stiffness increases as microgels become less viscous. In turn, these mechanical changes strongly influenced the mode of cell migration through the material.

Dr. Brendon Baker (University of Michigan, Ann Arbor, USA) described recent work from his lab focusing on the development of multicomponent hydrogels mimicking the biophysical and biochemical cues present within areas of interstitial tissues where idiopathic pulmonary fibrosis originates. The hydrogels, derived from a modified form of dextran, consisted of an interpenetrating network of electrospun fibers embedded within a bulk hydrogel with matrix metalloproteinase (MMP)-cleavable crosslinks. This tunable synthetic ECM enabled the Baker lab to determine how cues from the microenvironment drive myofibroblast differentiation and revealed that MMP activity is essential for myofibroblast differentiation in 3D, but not in 2D (Matera et al., 2020). Dr. Baker also demonstrated how the system can be used for screening of antifibrotic drugs.

Dr. Evangelia Bellas (Temple University, Philadelphia, USA) discussed the role of the ECM in regulating adipocyte fibrotic function using an engineered adipose tissue model consisting of differentiated adipocytes embedded within a crosslinked collagen hydrogel. This hydrogel system allows stiffness and architecture to be modified independently of the concentration of collagen. The Bellas Lab found that increased stiffness and altered architecture of the ECM result in a dysfunctional fibrotic state by inducing fibrotic gene expression and increasing the deposition of ECM. They also found that actin cytoskeletal stress fibers played an important role in cells sensing mechanical cues from the surrounding ECM (Caprio and Bellas, 2020).

Looking forward, engineered ECM microenvironments, such as the ones described here, can provide versatile systems to study the dynamic interplay between the ECM and cells in normal and pathologic 3D microenvironments, as well as drug screening platforms.

## Mentoring and career development at ASMB 2021

It is now more important than ever to train young scientific minds that will drive the future of biomedical research, clinical practice, and industrial innovation, while simultaneously communicating the importance of science, technology, engineering, and mathematics (STEM) to citizens and policymakers.

**Fig. 2.**
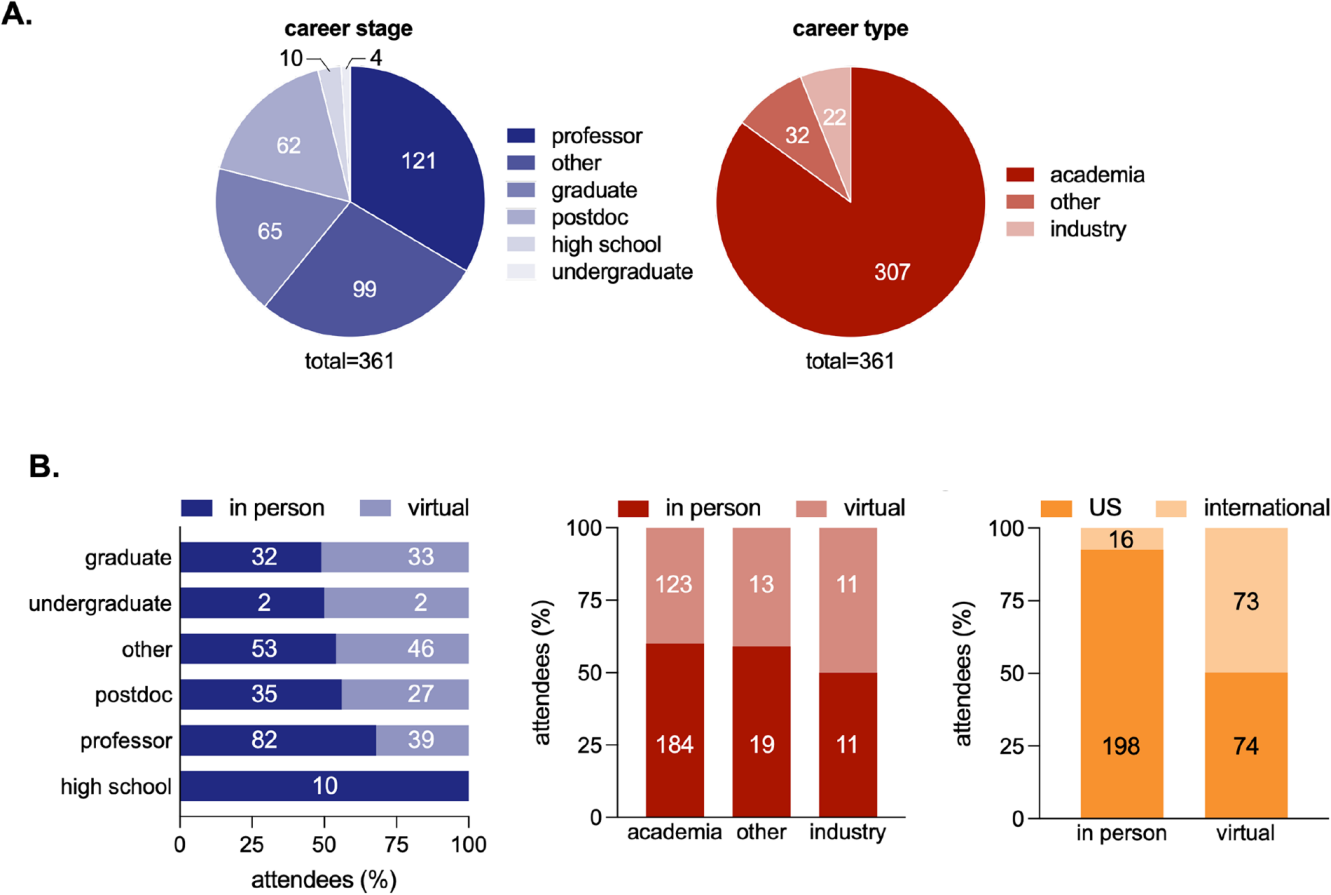
**Attendees of the 2021 ASMB hybrid scientific meeting.** (A) Breakdown of meeting participants by career stage (left panel) and sector (right panel). Both virtual and in-person attendees are included in this analysis. (B) Percentage of attendees that joined the conference in person or remotely based on career stage (left panel), sector (middle panel), and location (right panel).

To achieve such goals, the ASMB meetings have traditionally had a strong emphasis on promoting the science and careers of trainees and early-stage faculty members. This was also reflected during the 2021 meeting, where ∼70% of the attendees were trainees (Fig. 2A) and >50% of talks were presented by students and postdocs. A new flash-talk session allowed selected poster presenters to highlight their work. Several competitive awards were given by the ASMB, including eight travel awards based on submitted abstracts and five poster awards. Additional awards were presented by the Glaucoma Research Foundation and the Marfan Foundation. Finally, the first in-person Iozzo Trainee Award competition gave students and post-doctoral trainee finalists, selected by the ASMB Awards Committee, the opportunity to present their research to a large audience. The complete list of awardees can be found on the ASMB website.

Mentoring breakfasts offered a variety of discussion topics in an environment that allowed for open conversations between mentors and mentees. Topics included, ‘How to Make the Most of Graduate School’, ‘Non-Academic Careers’, ‘Defining Success in the Context of Work-Life Balance’, ‘Finding a Faculty Position’ and ‘Peer Review’. Unlike didactic discussions, group sessions enabled multiple viewpoints and multidisciplinary examination of these topics. These trainee-focused efforts were highly appreciated and will continue to play an important role in fulfilling the mission of the ASMB.

## New initiatives to enhance diversity, equity, and inclusion

The ASMB is committed to enhance diversity, equity, and inclusion (DEI) in the ECM community and, more broadly, in STEM fields. The 2021 scientific program strived to further this mission by increasing representation of women and underrepresented minority (URM) groups, as defined by the NIH (https://diversity.nih.gov/about-us/population-underrepresented). A post-meeting survey revealed that ∼50% of speakers and session chairs identified as women and 22% as URM. Additionally, 7% of the respondents identified as members of the LGBTQIA+ community, and 33% as first-generation college students. As in previous years, two career-mentoring breakfasts discussed topics including women in science, and promotion of DEI within the ASMB and the ECM research community. To further increase participation of URM trainees, the ASMB, for the first time, offered complimentary registration to 11 URM trainees. In addition, 30% of travel award recipients self-identified as URM.

In a special plenary session, Nikki Doughty, the Associate Director at the Institute for School Partnership at Washington University (https://schoolpartnership.wustl.edu), was invited to discuss City Academy, an independent St. Louis school that achieves a remarkable rate of 90% of graduating seniors entering tertiary education within STEM fields (https://cityacademystl.org). In addition, seven City Academy students were invited to the meeting and attended a special luncheon with members of the ASMB leadership and a poster session.

## Experience from a hybrid meeting and vision for the future

In the spring of 2021, the ASMB decided to move forward with the organization of a hybrid meeting. This decision was partly driven by previously established contracts with the venue and expiration of granted NIH funding. The choice of holding a hybrid meeting was made to allow the participation of domestic and international colleagues unable to travel due to restrictions related to the COVID-19 pandemic. Registration data indicate that of the 361 participants, 214 attended in person and 147 remotely. 198 of the 214 in-person participants (92.5%) were affiliated with U.S.-based institutions while 50% of the 147 remote attendees (73) were internationals (Fig. 2B). For comparison, the 2018 ASMB meeting counted 328 participants, including 255 (77%) affiliated to US institutions and 73 (22.3%) to international institutions.

The hybrid component of the meeting included live streaming of the five plenary sessions and 17 concurrent sessions. Remote attendees were also given the opportunity to present virtual posters and participate in mentoring breakfasts. Overall, integration of the in-person and virtual components went remarkably well. In addition, recordings of the sessions were made available to participants after the meeting and thus allowed on-site participants to watch concurrent sessions they missed.

As anticipated, the organization of its first hybrid meeting was a learning experience for the ASMB, and some organizational aspects will benefit from improvements. For example, session chairs sometimes missed the opportunity to engage with virtual attendees. In future iterations, we propose assigning a co-chair to specifically monitor online questions and engage with the virtual audience. Another challenging aspect was to engage remote attendees in the various networking opportunities, formal or informal. Despite being also offered online, these sessions suffered from low attendance. It was equally challenging to have remote poster presenters engaged during the on-site poster sessions. These aspects could and should be improved by more thoughtful consideration of time zone differences, more active advertising of virtual networking events and, in the case of poster sessions, having a dedicated live session in the program for a remote poster presenter live session equivalent to an on-site poster session.

On the matter of environmental responsibility, and as discussed elsewhere (Counsell et al., 2020), a hybrid meeting may substantially reduce the impact of meeting-related travels on the environment. Long- and short-distance air travel produce approximately ¼ ton CO_2_-equivalent per flight hour and even a short economy roundtrip from New York to St. Louis can produce 0.547 ton of CO_2_-equivalents per participant (https://co2.myclimate.org). And this does not consider additional travel to/from airports and accommodations. One innovative way for individuals to offset their accumulated carbon footprints would be by encouraging participants to donate to sustainability projects.

From a financial perspective, a cost-benefit analysis needs to account for how anticipated increased remote participation can offset increased costs due to advanced audiovisual (AV) and streaming service requirements. The total number of 361 participants for the ASMB 2021 hybrid meeting was comparable to attendance in previous years. However, AV costs increased by 70% and represented 23% of the total conference budget compared to about only 10% in previous years. The combination of increased AV costs and reduced registration fees for virtual attendees can make hybrid conferences more expensive and there may be a point where a hybrid option may cause financial challenges, especially for smaller scientific societies, such as the ASMB.

## Conclusion

The remarkable complexity of the ECM and the highly interdisciplinary nature of ECM research are two highlights of the 2021 meeting of the ASMB. The ECM is not only the structural scaffold it was once thought to be but is emerging as a critical contributor to cellular functions with implications in development, health, diseases, and for the development of therapeutic strategies.

On the organizational front, due to the unique advantages offered to both attendees and the ASMB, remote attending options will likely continue to play a role in advancing the missions of the ASMB. This will be especially relevant for participants who cannot attend meetings in person, due to work commitments, financial constraints, or challenges due to care giving responsibilities. Hybrid meetings, by breaking barriers, will permit a more global and faster dissemination of science and in turn will benefit the ECM research community.
